# Hardly Flammable Polyurethane Foams with 1,3-Pyrimidine Ring and Boron Atoms

**DOI:** 10.3390/polym13101603

**Published:** 2021-05-16

**Authors:** Elżbieta Chmiel-Szukiewicz

**Affiliations:** Department of Organic Chemistry, Faculty of Chemistry, Rzeszow University of Technology, al. Powstańców Warszawy 6, 35-959 Rzeszów, Poland; szukela@prz.edu.pl

**Keywords:** rigid polyurethane foams, flammability, thermal stability, 1,3-pyrimidine ring, 1,3-bis(2-hydroxyethyl)uracil, boric acid, ethylene carbonate

## Abstract

This work presents the results of research related to the determination of application possibilities of new oligoetherols with 1,3-pyrimidine rings and boron atoms in rigid polyurethane foam production. Oligoetherols were obtained from 1,3-bis(2-hydroxyethyl)uracil, boric acid, and ethylene carbonate. Their structure was determined by instrumental methods (IR, ^1^H-NMR and MALDI-ToF spectra) and the physicochemical and thermal properties were examined. Obtained oligoetherols were used for synthesis of polyurethane foams. Some properties of the foams, such as apparent density, water uptake, dimensions stability, thermal stability, compression strength, thermal conductivity, oxygen index, and horizontal burning were investigated. The introduction of boron atoms into the foam structure reduced their flammability, but unfortunately it had a negative effect on the water absorption of the obtained materials—the water absorption was higher compared to the boron-free foams. The obtained foams showed good thermal stability compared to classic, rigid polyurethane foams.

## 1. Introduction

Polyurethanes are polymeric materials which, thanks to their physicochemical properties, have been widely used in all areas of technology and economy. Among polyurethane plastics, foams are of great technological importance. They represent two thirds of the world’s production of polyurethanes [1]. The growing ecological awareness, combined with the size of polyurethane production, make it necessary to replace synthetic ingredients with natural or recycled ingredients. For this reason, there is a trend in the production of polyurethanes, including polyurethane foams, of using the polyols obtained from vegetable oils or from recycled products, e.g., polyethylene terephthalate [2,3,4,5,6,7,8,9,10]. Such foams have properties similar or better than the commercial foams available on the market. The foams based on bio-polyol are characterized by low water absorption (up to 2% after 24 h), low thermal conductivity (about 25 mW/(m × K)), and good compressive strength (up to 0.3 MPa) [2,8,9,10]. Good mechanical properties are demonstrated by the foams obtained from polyols based on rapeseed oil and recycled polyethylene terephthalate [9].

**Scheme 1:**
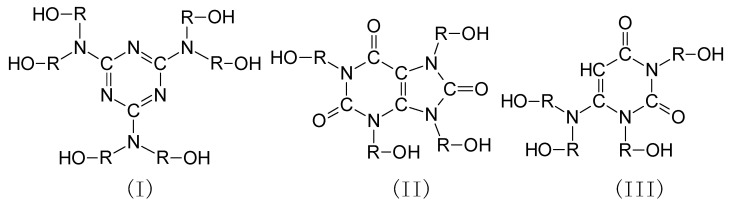
General structure of oligoetherols with 1,3,5-triazine (I), purine (II), and 1,3-pyrimidine (III) rings.

Polyurethane foam plastics are currently the most widely used insulating material. This is a result of their advantages, such as lower thermal conductivity coefficients than polystyrene and, additionally, they are light, durable, and easy to install. The main disadvantages of foams plastics are low thermal resistance and relatively low decomposition temperature. The thermal resistance of typical polyurethane foams usually does not exceed 120 °C [11], and the degradation of polyurethane foams is accompanied by the formation of flammable substances, which creates a fire hazard. In the course of burning, polyurethanes emit toxic gases, among others hydrogen cyanide, carbon monoxide and carbon dioxide, and nitrogen oxides [12,13]. This is a danger to human life and health, and for this reason methods to reduce the flammability of polyurethane foams and improve their thermal resistance are sought. Foams of improved thermal stability can be obtained by using an oligo- or polyetherol component containing some heterocyclic rings (Scheme 1), e.g., 1,3,5-triazine (I) [14,15], purine (II) [16,17], or 1,3-pyrimidine (III) [18,19,20,21].

Foams containing these heterocyclic rings in their structure are resistant to long-term heat exposure up to 200 °C, but unfortunately, they are flammable. This is a disadvantage, especially for thermal insulation materials that are to be used at high temperatures. Therefore, polyurethane foams are subjected to modifications increasing their fire resistance. One of the methods of reducing the flammability of polyurethane plastics consists in introducing atoms into the foam structure that impede the burning, e.g., nitrogen, phosphorus, silicon, or boron [22,23,24]. Currently, the use of halogen flame retardants is being abandoned due to their negative impact on people and the environment [25].

Quite common reactive flame retardants for polyurethane foams are organic esters of boric acid, which are polyol components in the synthesis of foams [26,27,28,29]. The use of oligo- and polyetherols with boron atoms and heterocyclic rings, e.g., perhydro-1,3,5-triazine or 1,3,5-triazine rings, allows obtaining foams with improved thermal stability and reduced flammability [30,31,32,33].

This paper proposes the syntheses of polyurethane foams with the use of oligoetherols obtained in the reactions of 1,3-bis(2-hydroxyethyl)uracil (BHEU) with boric acid (BA) and ethylene carbonate (EC). Two methods for the synthesis of oligoetherols are presented; their structures were determined and selected properties were examined. Properties of the foams obtained using the oligoetherols, such as thermal stability and flammability, were tested.

## 2. Materials and Methods

### 2.1. Synthesis of 1,3-bis(2-hydroxyethyl)uracil

BHEU was obtained from uracil (99%, Alfa Aesar, Germany) and ethylene oxide (pure, Honeywell-Fluka, Buchs, Switzerland) according to the patent [34].

### 2.2. Oligoetherols Synthesis

#### 2.2.1. Two-Step Synthesis of Oligoetherol

##### Reaction of BHEU with BA

First, 10.01 g (0.05 mol) of BHEU and 6.2 g (0.1 mol) of BA (pure, POCH, Gliwice, Poland) were placed in an open 100 cm^3^ three-necked flask, equipped with a mechanical stirrer and a thermometer. The contents of the flask were heated at 90–95 °C to melt the reactants, then 1 cm^3^ of distilled water was added to better homogenize the reactants. Then, the stirrer was started and the open flask was heated at 120 °C for about 30–35 min, until the appropriate weight loss associated with evaporation of the water formed in the reaction was reached. The obtained product (2,4-dioxopyrimidine-1,3-diethyl bis(dihydroborate), DOPDEDHB, elemental analysis: found: 32.93% C, 4.97% H, 9.55% N; calculated: 33.39% C, 4.90% H, 9.74% N) was a creamy, glassy mass. 

##### Reactions of DOPDEDHB with EC

To a three-necked flask equipped with a mechanical stirrer, reflux condenser and thermometer, containing 14.41 g (0.05 mol) of DOPDEDHB, 52.84 g (0.6 mol) or 70.45 g (0.8 mol) of EC (pure, Honeywell-Fluka, Buchs, Switzerland) and 1 g of potassium carbonate as catalyst were added. After the reactants were melted (90–95 °C), the stirrer was started and the mixture was heated at 160 °C for 19 (0.6 mol EC) or 33 h (0.8 mol EC). The end of the reaction was determined by analyzing the IR spectrum of the reactions mixture—the disappearance of the band at 1800 cm^−1^ of valence vibrations of carbonyl group C=O of unreacted EC was used as the indicator.

#### 2.2.2. One-Pot Synthesis of Oligoetherol

To a three-necked flask equipped with a mechanical stirrer, reflux condenser and thermometer 10.01 g (0.05 mol) of BHEU, 6.2 g (0.1 mol) or 9.3 g (0.15 mol) of BA and 52.84 g (0.6 mol) of EC were added. The contents of the flask were melted at 90–95 °C, then the stirrer was started and the mixture was heated for 4 h at 120 °C. After this time, 1 g potassium carbonate was added, the temperature was increased to 160 °C and the mixture was heated for 38 (0.1 mol BA) or 28 (0.15 mol BA) hours. The end of the reaction was determined by analyzing the IR spectrum of reactions mixture—the disappearance of the band at 1800 cm^−1^ of valence vibrations of carbonyl group C=O of unreacted EC was used as the indicator.

### 2.3. Foams Synthesis

A 10 g quantity of oligoetherol was introduced into a 500 cm^3^ polypropylene cup, then 1.95% silicone L-6900 (pure, Momentive Performance Materials, Wilton, CT, USA) as a surfactant, 2–3% distillate water, and 2.16–4.86% TEA (pure, Avantor Performance Materials Poland S.A., Gliwice, Poland) as a catalyst were added. The mixture was vigorously stirred and then polymeric 4,4′-diphenylmethane diisocyanate (pMDI, mixture of di- and triisocyanates (30%) for synthesis; Merck-Schuchardt, Hohenbrunn, Germany) was added (Table 1). The mixture was vigorously stirred until creaming started.

### 2.4. Analytical Methods

The infrared spectra were registered on ALPHA FT-IR spectrometer, (Bruker, Ettlingen, Germany) ATR technique. The ^1^H-NMR spectra were recorded with a 500 MHz Bruker UltraShield spectrometer in d_6_-DMSO with hexamethyldisiloxane as internal standard. Elemental analyses for C, H, and N of the product reaction of BHEU with BA were done with Vario EL III, Elementar Analyzer (Elementar, Langenseldbold, Germany). MALDI-ToF spectra (matrix-assisted laser desorption/ionization time of flight) were obtained on a Bruker autoflex speed reflectron time-of-flight mass spectrometer (Bruker, Ettlingen, Germany), equipped with a SmartBeam II laser (Bruker, Ettlingen, Germany) (352 nm) in 80–2000 m/z range. Mass calibration was performed using internal standards (gold ions and clusters from Au^+^ to Au_10_^+^ depending on m/z range). The sample solution (ca. 5 mg/mL in H_2_O) was placed on AuNPET [35] (0.5 μL) with a 0.5 μL standard α-cyano-4-hydroxycinnamic acid solution (1:1 water:acetonitrile with 0.2% TFA). The oligoetherols were also analyzed by gas chromatography with a Hewlett–Packard 4890A instrument (Agilent Technologies Deutschland GmbH, Waldbronn, Germany) equipped with a flame ionization detector. From chromatograms, the amounts of side products (glycols and polyglycols) formed in the reaction were determined. The gas chromatography conditions were as follows: HP-FFAP capillary column of 30 m length, 0.53 mm diameter, 1.5 μm film thickness, port temperature: 220 °C, temperature profile: 50–220 °C, heating rate 20 deg./min, the helium flow 18.3 cm^3^/min, and 0.2 μdm^3^ sample volume. The calibration was performed with cyclohexanone (≥99%, S.A. POCH, Gliwice, Poland) as an internal standard. A series of reference substances were used: ethylene glycol, diethylene glycol, triethylene glycol, and tetraethylene glycol, (pure Aldrich, UK). The percentages of glycols/polyglycols in products were determined based on calibration curves with the same internal standard using Equation (1):

(1) $$\frac{m_{g}}{m_{st}}=a\times \frac{S_{g}}{S_{st}}+b$$

where: *m_g_, m_st_*: glycol/polyglycol mass and mass of standard, respectively; *S_g_, S_st_*: integrated peak area of glycol/polyglycol and standard, respectively; *a, b*: experimental coefficients of calibration curves.

The mass of side products (glycols and polyglycols) were calculated from Formula (1). The amount of side products in mass percentage were calculated considering total sample mass (*m_s_*) according to Equation (2):

(2) $$\%G=\frac{m_{g}}{m_{s}}\times 100\%$$

Some other properties of the oligoetherols were evaluated, such as density (pycnometrically), viscosity (Höpler viscometer, type BHZ, VEB Prüfgerate-Werk Medingen, Freital, Germany), and surface tension, by the detaching ring method. The surface morphologies of foams were photo-recorded with Malvern’s MORPHOLOGI G3 apparatus (Malvern Instruments Ltd., Malvern, UK)with 123 (zoom 2.5) and 247 (zoom 5.0) enhancement lenses. The thermal analysis of foams was conducted with a thermogravimetric analyzer TGA/DSC 1 (Mettler Toledo, Spain); the recording conditions were as follows: sample weight 2–4 mg, temperature range 25–600 °C, recording time 60 min, and nitrogen atmosphere. Thermal resistance of foams was determined also by static method. The foams were heated for a month (during this time the foam mass stabilizes) at 150, 175, and 200 °C with continuous measurement of mass loss. Mass loss of foam was calculated from Equation (3):

(3) $$\Delta m=\frac{m_{0}-m_{t}}{m_{0}}\times 100\%$$

where: *m*_0_, *m_t_*: mass of the sample before and after heated, respectively (g).

The apparent density of foams (the ratio of foam weight to its geometrical volume) was determined for cube-shaped samples according to the norm [36]. Water absorption was tested according to the norm [37] by immersing the samples in distilled water for 5 min, 3 h, and 24 h. Water absorption in vol % was calculated from Equation (4):

(4) $$\%W_{A}=\frac{m_{2}-m_{1}}{V_{0}\times d_{W}}\times 100\%$$

where: *m*_1_, *m*_2_: mass of the sample before and after immersion in distilled water, respectively (g), *V*_0_: volume of the sample before immersion in distilled water (cm^3^), d_W_: density of distilled water (*d_W_* = 1 g/cm^3^).

Linear dimension stability of foams was determined according to the norm [38] by thermostating the samples at a temperature 150 °C for 20 and 40 h and measuring changes in length (*Δl*), width (*Δb*), and thickness (*Δδ*) of samples (Equations (5)–(7)):

(5) $$\Delta l=\frac{l_{1}-l_{0}}{l_{0}}\times 100\%$$

(6) $$\Delta b=\frac{b_{1}-b_{0}}{b_{0}}\times 100\%$$

(7) $$\Delta \delta =\frac{\delta_{1}-\delta_{0}}{\delta_{0}}\times 100\%$$

where: *l*_0_, *b*_0_, *δ*_0_, *l*_1,_ *b*_1_, *δ*_1_: dimensions of samples before and after thermostating, respectively (mm).

Thermal conductivity of foams was determined by measuring the thermal conductivity coefficient (λ) with IZOMET 2104 (Bratislava, Slovakia) according to the norm [39]. The compressive strength was determined using the testing machine with an electronic head FT 100 (Heckert, Chemnitz, Germany) according to the norm [40]. The maximum force and relative strain (decreasing of the height of foam in relation to the initial height, in accordance with the direction of foam rise) or maximum force inducing 10% relative strain foams before and after exposition at 150 °C were determined. Horizontal burning test was determined according to the norm [41]. The samples (150 mm × 50 mm × 13 mm) were weighed and located on stainless steel net (200 mm × 80 mm). The line on every sample at the distance of 25 mm from the edge was marked. The sample was set on fire from the bottom using a Bunsen burner with the blue flame of 38 mm height for 60 s. Then the burner was removed and time of free burning of foam by stopwatch was measured from when the flame or glowing combustion front passed the 25 mm gauge mark. After the test, the samples were weighed again. The rate of burning (*v*) was calculated from the Equation (8):

(8) $$v=\frac{L_{e}}{t_{b}}$$

where: *L_e_*: the distance burnt (between the 25 mm gauge mark and the point where the flame or glowing combustion front stopped, mm), *t_b_*: burning time (s).

The mass loss (Δ*m*) after burning was calculated according to the Equation (9):

(9) $$\Delta m=\frac{m_{0}-m}{m_{0}}\times 100\%$$

where: *m*_0_, *m*: mass of the sample before and after burning, respectively (g).

Oxygen index was measured with Concept Equipment apparatus (Concept Equipment, Rustington, UK) according to the norm [42]. In the mixture of oxygen and nitrogen, the percentage-limited concentration of oxygen, sufficient to sustain the burning of the sample, was determined. Oxygen index (*LOI*) was calculated from Equation (10):

(10) $$LOI=\frac{\left[O_{2}\right]}{[O_{2}]+[N_{2}]}\times 100\%$$

where: [*O*_2_], [*N*_2_]: volumetric flow of oxygen and nitrogen at the limit concentration, respectively (m^3^/h).

## 3. Results and Discussion

### 3.1. Oligoetherols Synthesis and Properties

**Scheme 2:**
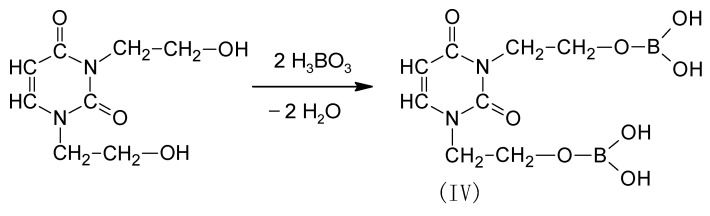
Reaction of 1 mol BHEU with 2 moles BA.

Oligoetherols with 1,3-pyrimidinering were obtained in two ways. In the first method, reaction of BHEU with BA was carried out (Scheme 2).

**Scheme 3:**
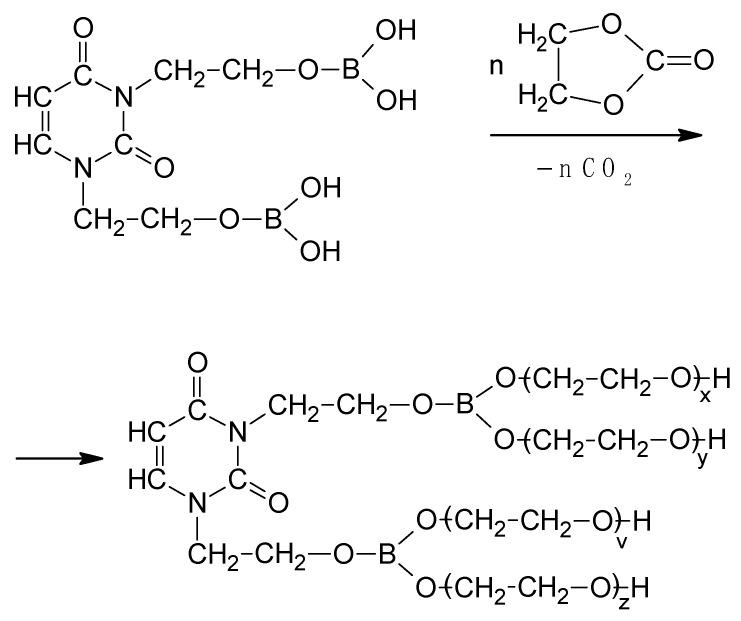
Reaction of DOPDEDHB with an excess of EC, where: *n* = x + y + z = 12 or 16.

The next obtained product (DOPDEDHB, IV) was treated with excess of EC in the presence of potassium carbonate (Scheme 3).

The syntheses were carried out at 160 °C, with a molar ratio of DOPDEDHB to EC equal to 1:12 and 1:16. The amount of EC was selected to obtain an oligoetherol with a high boron content and with a consistency that allows mixing it with the isocyanate (if a smaller amount of EC was added, oligoetherols of large density at room temperature were obtained). The course of the reaction and its end were determined by analyzing the IR spectrum of reactions mixture—the disappearance of the band at 1800 cm^−1^ of valence vibrations of carbonyl group C=O of unreacted EC was used as the indicator. Dark brown, thick, resin-like, soluble-in-water products were obtained. The structure of the obtained oligoetherols was determined by instrumental methods (IR, ^1^H-NMR and MALDI-ToF spectra).

**Figure 1:**
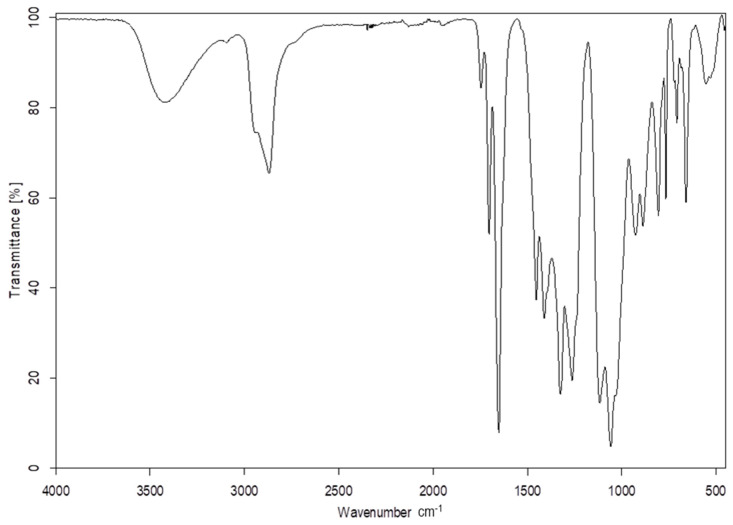
IR spectrum of the product of reaction of 1 mol DOPDEDHB with 12 moles EC.

In the IR spectra of the products of the reaction of DOPDEDHB with excess EC (Figure 1) the following were present: a valence vibration band of hydroxyl groups at 3427 cm^−1^; the asymmetric and symmetric valence vibration bands and the deformation vibration bands of methylene groups at 2931, 2870, and 1454 cm^−1^, respectively; valence vibration bands of carbonyl groups and of unsaturated bond in the 1,3-pyrimidine ring at 1703 and 1656 cm^−1^, respectively; valence vibration bands of the B–O bond at 1411 and 1326 cm^−1^; valence vibrations of ether groups (C–O–C) at 1118 cm^−1^; and C–O bonds in primary alcohols at 1060 cm^−1^. The bands at 928, 766, and 549 cm^−1^ were attributed to vibration of the 1,3-pyrimidine ring. In the IR spectra of oligoetherols, there was also a band at 1746 cm^−1^, derived from the valence vibrations of the ester groups (–CH_2_–O–CO–O). This indicates that the reaction took place to a small extent with preservation of the carbonate group.

**Figure 2:**
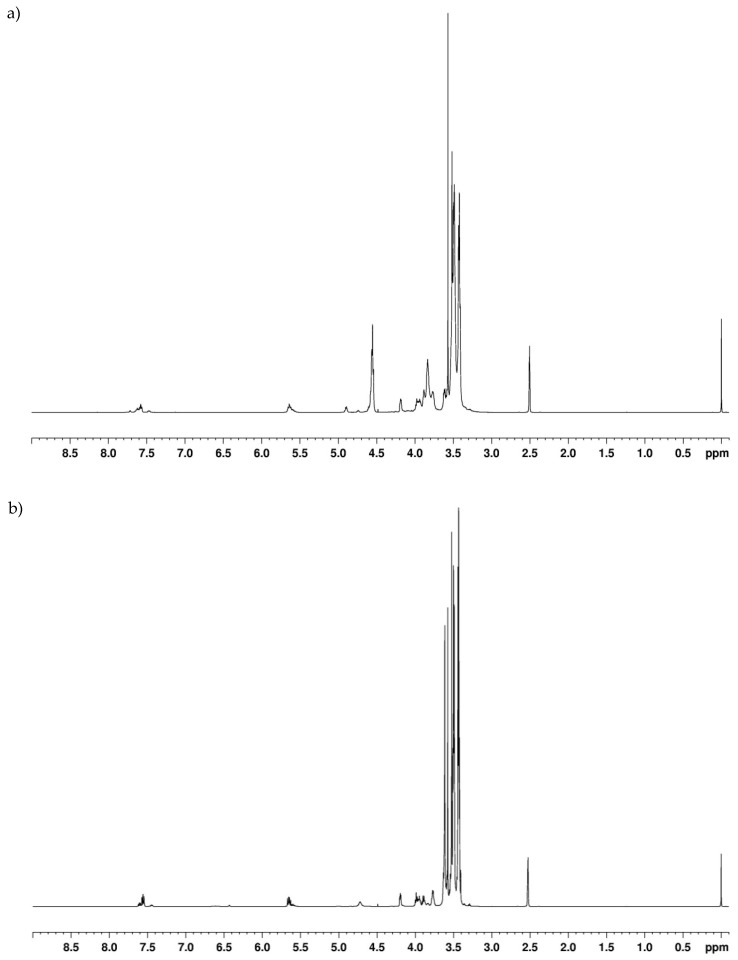
^1^H-NMR spectrum of the product of reaction of 1 mol DOPDEDHB with 12 moles EC (**a**), with D_2_O (**b**).

In the ^1^H-NMR spectra of the obtained oligoetherols (Figure 2), signals of methylene protons in oxyethylene groups (–O–CH_2_–CH_2_–O–) and borate groups (–CH_2_–O–B) at a chemical shift in the range of 3.4–3.7 ppm and the ester groups (–CH_2_–O–CO–O) at 4.18 ppm were observed. The intensity of these signals increased as the amount of EC used in the reaction increased. The signals of protons connected with unsaturated carbon atoms in the ring =C(5)–H and =C(6)–H were located at a chemical shift of 5.64 and 7.58 ppm, respectively. The splitting of the signal of the proton of carbon C(6) in the ring was associated with various atomic surrounding of the proton. Signals of hydroxyl protons appeared in the range of 3.7–5 ppm and overlapped with the signals of methylene protons, connected with nitrogen atoms (>N–CH_2_–CH_2_–O–, 3.7–4 ppm), which was confirmed by the spectra with D_2_O (Figure 2a,b). Since, after D_2_O addition, the signal at 4.75 ppm had not disappeared completely, it can be concluded that unsaturated bonds were present in the reactions products. Unsaturated bonds formed at the ends of chains as a result of H_2_O elimination in the high-temperature process (160 °C). Their amount was not significant. In the spectra a characteristic signal at 3.57 ppm was also visible, indicating the presence of 1,4-dioxane in the product. This compound was formed at high temperature from ethylene glycol. Ethylene glycol was formed in the side reaction of EC with water contained in the hydroborate.

Therefore, a chromatographic analysis was performed to identify and evaluate the content of by-products of ethylene glycol and the products of its consecutive reactions with EC. An analysis of postreaction mixtures indicated presence of ethylene glycol and tetraethylene glycol in amounts: 1.28% and 5.78% in the product of reaction of DOPDEDHB with 12 moles EC, and 0.89% and 7.49% in product of reaction of DOPDEDHB with 16 moles EC, respectively. Analysis of the MALDI-ToF spectra of products of reactions of DOPDEDHB with EC indicated the formation of oligoetherols with different oxyethylene chain lengths (Table 2). In spectra, the peaks of molecular ions differing by M/z = 44.05 (oxyethylene sub-units) could be observed. Since the analysis of the IR and ^1^H-NMR spectra of the oligoetherols indicated presence of ester groups, structures containing a carbonate groups were also proposed (the molar mass of oxyethylene and carbonate groups is the same). The spectra showed that unsaturated structures formed. Many of the unsaturated structures were generated in the conditions of registration of the MALDI-ToF spectrum (high temperature favors the elimination of water from the hydroxyethyl groups). Analysis of the ^1^H-NMR spectra with D_2_O showed that a very small amount of unsaturated structures were present. The occurrence of molecular peaks with mass increased by the unit M/z = 39, was related to the presence of potassium cations from the catalyst used (potassium carbonate). The spectrum also confirmed the formation of 1,4-dioxane and glycols (Table 2, No. 1–3).

**Scheme 4:**
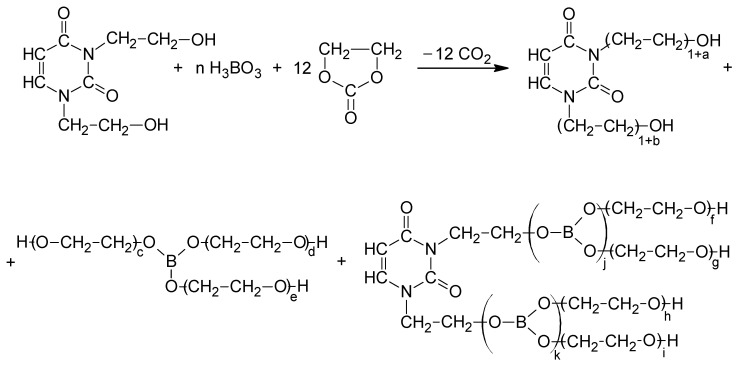
Reaction of BHEU with BA and EC, where: *n* = 2 or 3, a + b + c + d + e + f + g + h + i = 12, j + k < 2 or 3.

The second method of synthesis of oligoetherols relied on the direct reaction of BHEU with BA and EC (Scheme 4).

**Figure 3:**
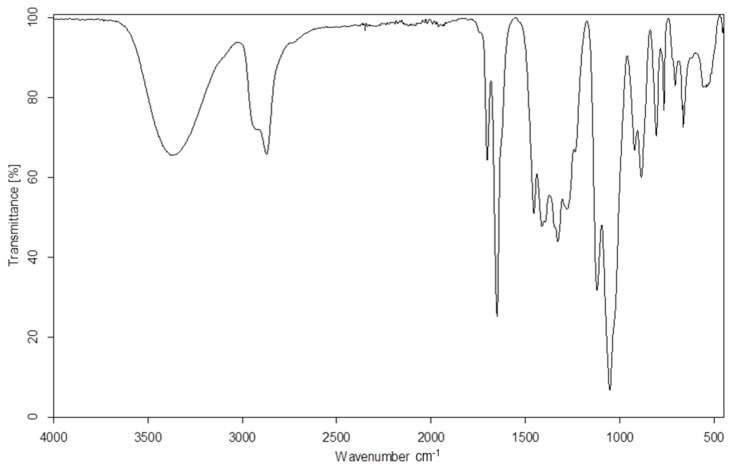
IR spectrum of the product of reaction of 1 mol BHEU with 2 moles BA and 12 moles EC.

The syntheses were carried out at an initial molar ratio BHEU, BA, and EC equal to 1:2:12 and 1:3:12. The mixture was heated at 120 °C for 4 h. This temperature allowed the start of the esterification of BHEU with BA and the reaction of BA with EC [31]. After this time, potassium carbonate (catalyst) was added and the temperature was increased to 160 °C. This temperature allowed the start of the reaction of EC with hydroxyethyl and hydroborate groups. The end of the reaction, as before, was determined by analyzing the IR spectrum of the reactions mixture. Dark brown, liquid, resin-like, soluble in water products were obtained. The structure of the obtained oligoetherols was determined by instrumental methods (IR, ^1^H-NMR and MALDI-ToF spectra). The IR spectra of the reaction products of BHEU with BA and EC (Figure 3) and the reaction products of DOPDEDHB with EC (Figure 1) were similar. In the spectra of the reactions products of BHEU with BA and EC (Figure 3), the presence of the band from the valence vibrations of the ester groups at 1746 cm^−1^ was not found only.

**Figure 4:**
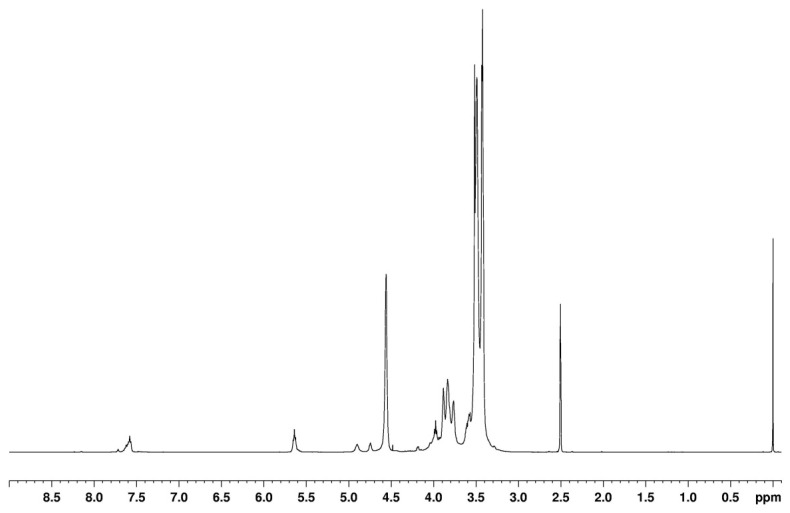
^1^H-NMR spectrum of the product of reaction of 1 mol BHEU with 2 moles BA and 12 moles EC.

**Figure 5:**
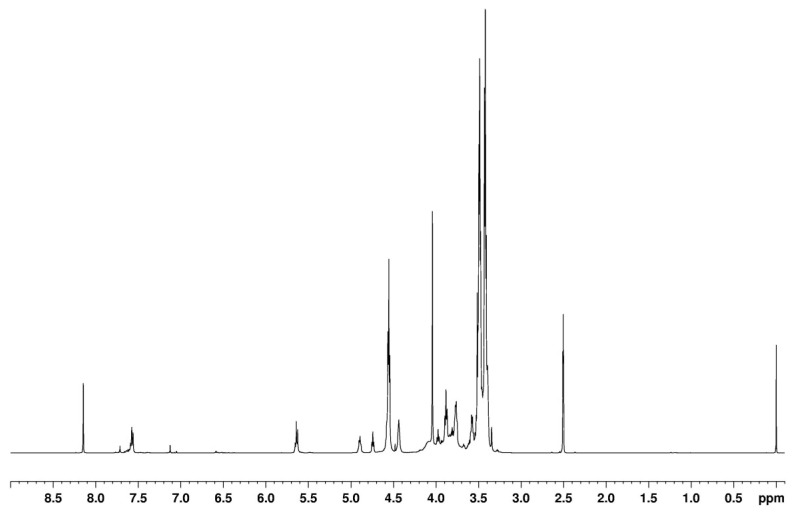
^1^H-NMR spectrum of the product of reaction of 1 mol BHEU with 3 moles BA and 12 moles EC.

Also, the ^1^H-NMR spectra of the reaction products of BHEU with BA and EC (Figure 4 and Figure 5) were similar to the spectra of the products obtained in the reaction of DOPDEDHB with EC (Figure 2a). In the spectra, however, signals of methylene protons in ester groups were not observed. The use of 3 moles of BA in the reaction resulted in the presence in the spectrum (Figure 5) of small signals of protons of hydroborate groups (7.12 ppm) and of unreacted BA (8.14 ppm).

Chromatographic analysis of postreaction mixtures indicated presence 3.03% of ethylene glycol and 2.89% of tetraethylene glycol in product of reaction of BHEU with 2 moles BA and 12 moles EC and 4.22% of triethylene glycol in product of reaction of BHEU with 3 moles BA and 12 moles EC. The MALDI-ToF spectra of oligoetherols (Table 3) confirmed the occurrence of the reactions presented in Scheme 4. The peaks corresponding to the products of reaction of BA with EC (Table 3, Nos. 3, 4, 6, 7, 9, 10, 12–15), BHEU with EC (Table 3, Nos. 5, 8, 10,11, 13, 15–21), 1-(2-hydroxyethyl)-2,4-dioxopyrimidine-3-ethyl dihydroborate/3-(2-hydroxyethyl)-2,4-dioxopyrimidine-1-ethyl dihydroborate (HEDOPEDHB), and DOPDEDHB with EC (Table 3, Nos. 11, 13, 15–21) were present. There were also unsaturated structures in the spectra, with the mass reduced by the unit M/z = 18.

The physical properties of oligoetherols were examined. Measured viscosity, density, and surface tension values (Table 4) showed that these obtained compounds are suitable substrates for obtaining polyurethane foams.

### 3.2. Polyurethane Foams Synthesis

In the next stage, the obtained oligoetherols as polyol components to prepare polyurethane foams were used. Foaming was carried out in a laboratory scale. The composition of foaming samples was selected experimentally. As a catalyst, triethylamine was used, and as a surfactant, silicone L-6900 was applied. It was found that foams with regular, small pores are obtained using 3 wt% of water, 2.7–3.85 wt% of catalyst, 1.95 wt% of surfactant and 140–160 g of isocyanate per 100 g of oligoetherol (Table 1). For this composition of the reaction mixture, the creaming time was in the range of 27–46 s, the expanding time was 9–17 s, and the foams immediately dried (Table 1).

**Figure 6:**
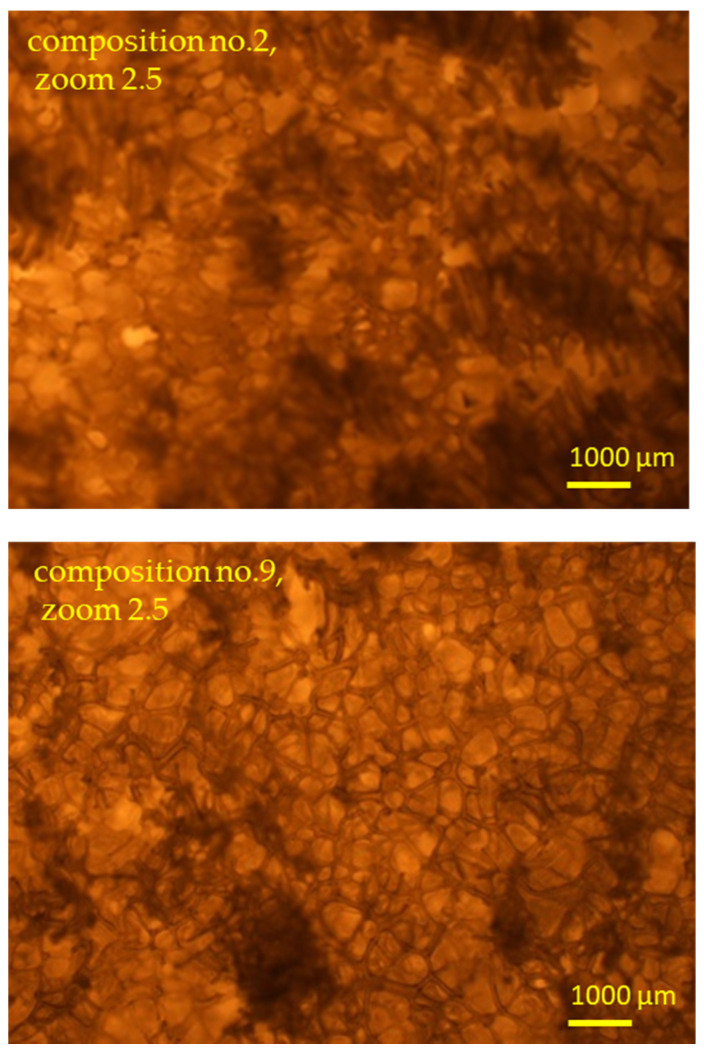

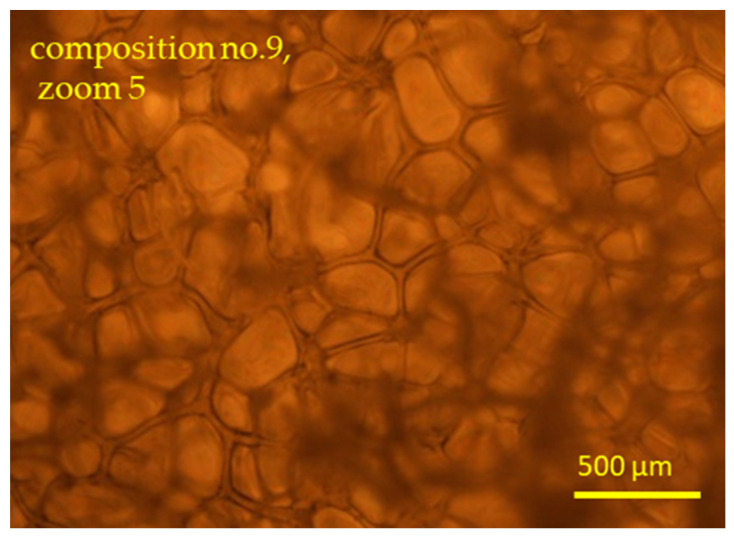
Microscope image of the foams.

Based on digital images of foams (Figure 6) one can notice that foams obtained from oligoetherols synthesized by the two-step method did not differ much from foams based on oligoetherols obtained in the direct reaction of BHEU with BA and EC. Digital images of compositions no. 2 and 9 (Figure 6) showed that the pore diameter was between 300 and 600 μm.

### 3.3. Properties of Foams

The physical properties—apparent density, water uptake, dimension stability, compression strength, thermal conductivity coefficient, thermal resistance, and flammability—of selected polyurethane foams were studied. 

The apparent densities of the foams were in the range of 56–91 kg/m^3^ (Table 5), so they were classified as rigid materials. Along with the increase in the content of boron in foams, their apparent densities increased. The highest density (91 kg/m^3^) showed the foam synthesized from oligoetherol obtained in the direct reaction of BHEU, BA, and EC with the initial molar ratio of the reactants equal to 1:3:12. The obtained foams had a higher apparent density than the foams based on oligoetherols synthetized from 6-aminouracil (AU), EC, and propylene oxide (PO) (Table 5).

Water uptake of the obtained foams (Table 5) was significantly larger than one of the foams without boron incorporated, prepared from oligoetherols synthesized in reactions of AU with EC and PO [20]. Water absorption after 24 h of exposition was between 8.05–27.62 vol%, the lowest uptake (8.05 vol%) characterized the foam based on product of reaction of 1 mole of DOPDEDHB with 16 moles EC (Table 5). Increased water absorption of obtained foams was due to the presence of boron and the formation of a coordination bond between the water oxygen atom and the boron atom.

Dimensional stability tests showed that shrinkage of the foams after 40 h exposure at 150 °C is small (Table 5). The largest shrink was observed for composition no. 2, and the smallest for composition no. 4. The foams without boron atoms showed both positive and negative dimensional changes (Table 5).

**Figure 7:**
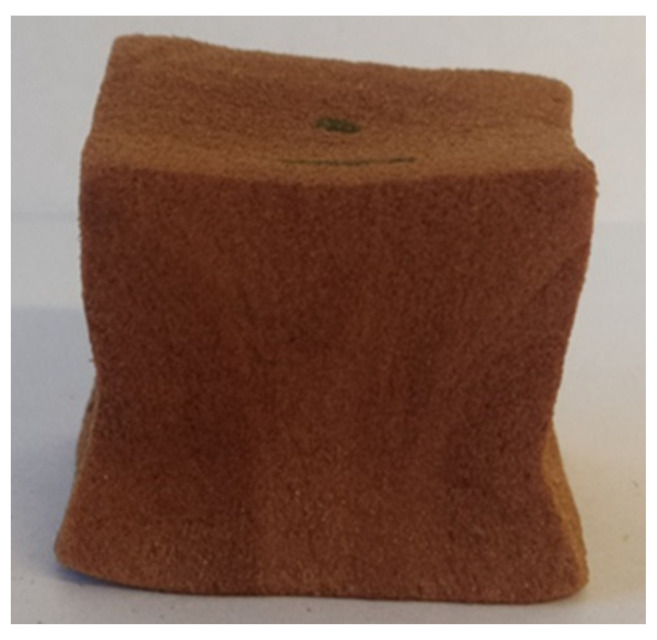
Composition 7 after exposition at temperature 175 °C.

The thermal resistance of foams was determined by registering the loss of mass of compositions at 150 °C, 175 °C, and 200 °C (Table 6). The samples were exposed to thermal treatment for one month, because the foams reach a constant mass during this time. After 4 h of heating at 200 °C, all compositions showed a significant loss of mass, very high shrinkage, and deformation, therefore the tests at this temperature were not continued. All samples showed distortion of their shape after 30 days of thermal treatment at temperature 175 °C (Figure 7), and after 30 day of thermal treatment at 150 °C only sample No. 4 showed distortion.

The gradual loss of mass was observed, but the highest loss of mass of foams was always observed after the first day of exposition. Stabilization of mass was reached after 23–25 days. The mass losses of foams after exposition at temperatures of 150 °C and 175 °C for 30 days were 16–24 wt.% and 34–38 wt.%, respectively (Table 6). All foams after thermal treatment became more rigid. Dynamic thermal analysis showed that 10% mass loss was observed at temperature range 202–235 °C and 50% mass loss at 299–322 °C (Table 6). The research indicates that polyurethane foams prepared from oligoetherols synthesized from BHEU, BA, and EC are characterized by better thermal resistance than classic industrial polyurethane foams and worse than foams based on oligoetherols from AU and EC or from AU, EC, and PO (Table 6).

The thermal conductivity coefficients of the obtained materials fell in the range 0.0349–0.0368 W/(m × K) (Table 7). These were larger in comparison with typical rigid polyurethane foams (0.019–0.026 W/(m × K)) [10], but still indicate good thermal insulation properties of boron-modified foams.

Mechanical properties were evaluated on the basis of compression strength measurements (Table 7). Before the thermal treatment, compression strengths of the foams were in the range of 0.29–0.33 MPa. It has been observed that one-month-long thermal treatment at temperature 150 °C resulted in higher compression strengths (0.58–1.27 MPa). This is presumably related to structural changes of polyurethane foams. During exposition at the temperature of 150 °C, the process of additional cross-linking of the foams probably occurred, and the material degradation had not taken place yet. The foam based on oligoetherol obtained from DOPDEDHB and 16 moles of EC was characterized by the highest compressive strength (1.27 MPa) after exposition at 150 °C. The tested foams resisted significantly higher forces than the foams based on obtained oligoetherol from AU and 6 moles EC (0.14 MPa) [19].

Flammability of the foams was determined by the methods of horizontal burning test and oxygen index (Table 8). The horizontal burning test showed that all the foams were self-extinguishing in the air. In the horizontal test, the flame reached merely in the range 11–20 mm from the ignition start point, and the flaming rate was in the range 2.06–3.42 mm/s (Table 8). Mass losses during burning amounted to 3.81 wt% (composition No. 4) up to 4.97 wt.%. (composition No. 9). The oxygen index of the obtained foams had values in the range of 22.0–24.1 vol% (Table 8) The limit value of the oxygen index (LOI) distinguishes three classes of flammability of materials: flammable—LOI < 21%, fire retardant—LOI = 21–28%, and nonflammable—LOI > 28% [1]. Thus, the obtained foams can be classified as class two. The foams with the highest boron content (composition No. 4) presented the lowest flammability. The flammability tests indicated that the obtained polyurethane foams with 1,3-pyrimidine ring and boron atoms are self-extinguishing and flame-retardant [43].

Polyurethane foams obtained from oligoetherols synthesized from 1,3-bis(2-hydroxyethyl)uracil, boric acid, and ethylene carbonate characterized a reduced flammability and improved thermal stability compared to classic, rigid polyurethane foams.

## 4. Conclusions

Oligoetherols obtained from 1,3-bis(2-hydroxyethyl)uracil, boric acid, and ethylene carbonate are suitable for manufacturing of polyurethane foams with improved thermal stability and reduced flammability. The obtained polyurethane foams have better thermal resistance than the resistance of classic polyurethane foams and can be used at a temperature range of 150–175 °C. Total mass losses of 10% were observed in the foams in the temperature range of 202–235 °C. The water absorption rates of these foams were larger than the water absorption of commercial foams and after 24 h of exposition they were within the range 8.05–27.62 vol%. This fact is due to the presence of boron and the formation of a coordination bond between the water oxygen atom and the boron atom. The other properties of foams, such as, apparent density, dimensions stability, thermal conductivity, and compression strength were similar to the properties of the commercial polyurethane foams. The apparent density rates of the foams were in the range of 56–91 kg/m^3^, the thermal conductivity levels fell in the range 0.0349–0.0368 W/(m × K), compression strength values were in the range of 0.29–0.33 MPa. The compression strengths of foams after exposition at temperature 150 °C for 30 days increased to 0.58–1.27 MPa. The oxygen indexes of the foams were in the range of 22.0–24.1 vol%, hence the obtained materials were hardly flammable under normal atmospheric conditions, in contrast to foams based on oligoetherols obtained from AU and EC or from AU, EC, and PO, without the addition of boron.

## Figures and Tables

**Table 1:** The influence of composition on foaming process.

Oligoetherol—Product of Reaction	Composition Number	Composition * (g/100 g of Oligoetherol)			Foaming Process			Characteristics of Freshly Prepared Foams
		pMDI	TEA	Water	Time of Creaming (s)	Time of Expanding (s)	Time of Drying (s)	
BHEU:BA:EC 1:2:12	1	128	2.16	2	52	10	1	insufficiently grown, hard
	2	149	2.70	3	46	17	1	small shrink, small regular pores, rigid
BHEU:BA:EC 1:3:12	3	165	4.31	3	40	12	5	rigid after time
	4	160	3.77	3	43	9	1	rigid
DOPDEDHB:EC 1:12	5	80	3.23	3	35	60	190	unrigid
	6	120	4.86	3	25	43	1	small shrink, small regular pores, rigid
	7	140	3.85	3	32	20	1	small regular pores, rigid
DOPDEDHB:EC 1:16	8	120	4.31	3	79	23	1	rigid after time
	9	140	3.50	3	27	11	1	small regular pores, rigid

* a constant amount of the surfactant (silicone) was used at 1.95/100 g oligoetherol.

**Table 2:** Interpretation of MALDI-ToF spectrum of the product of reaction of 1 mol DOPDEDHB with 12 moles EC.

No	Signal Position (M/z)	Probable Structure of Molecular Ion	Calc. Molecular Weight (g/mol)
1	83.1	Ethylene glycol + K^+^ − H_2_O	83.0
2	89.1	1,4-dioxane + H^+^	89.1
3	233.1	Tetraethylene glycol + K^+^	233.1
4	397.3	DOPDEDHB + 2 OE − H_2_O + K^+^ DOPDEDHB + OE − H_2_O + CO_2_ + K^+^	397.1 397.1
5	415.1	DOPDEDHB + 2 OE + K^+^	415.1
6	421.1	DOPDEDHB + 3 OE + H^+^ DOPDEDHB + 2 OE + CO_2_ + H^+^	421.1 421.1
7	459.2	DOPDEDHB + 3 OE + K^+^ DOPDEDHB + 2 OE + CO_2_+ K^+^	459.1 459.1
8	465.2	DOPDEDHB + 4 OE + H^+^ DOPDEDHB + 3 OE + CO_2_+ H^+^	465.2 465.2
9	503.2	DOPDEDHB + 4 OE + K^+^ DOPDEDHB + 3 OE + CO_2_+ K^+^	503.2 503.2
10	509.2	DOPDEDHB + 5 OE + H^+^ DOPDEDHB + 4 OE + CO_2_+ H^+^	509.2 509.2
11	534.3	DOPDEDHB + 6 OE − H_2_O DOPDEDHB + 5 OE − H_2_O + CO_2_	534.2 534.2
12	553.3	DOPDEDHB + 6 OE + H^+^ DOPDEDHB + 5 OE + CO_2_+ H^+^	553.3 553.3
13	579. 4	DOPDEDHB + 7 OE − H_2_O + H^+^ DOPDEDHB + 6 OE − H_2_O + CO_2_+ H^+^	579.3 579.3
14	597.3	DOPDEDHB + 7 OE + H^+^ DOPDEDHB + 6 OE + CO_2_+ H^+^	597.3 597.3
15	641.2	DOPDEDHB + 8 OE + H^+^ DOPDEDHB + 7 OE + CO_2_+ H^+^	641.3 641.3
16	773.5	DOPDEDHB + 11 OE + H^+^ DOPDEDHB + 10 OE + CO_2_+ H^+^	773.4 773.4

where: DOPDEDHB is a fragmentation product of 2,4-dioxopyrimidine-1,3-diethyl bis(dihydroborate), OE is an oxyethylene group, CO_2_ is a carbonate group, and the number preceding the symbol indicates the number of oxyethylene groups in a structure.

**Table 3:** Interpretation of MALDI-ToF spectrum of the product of reaction of 1 mol BHEU with 2 moles BA and 12 moles EC.

No	Signal Position (M/z)	Probable Structure of Molecular Ion	Calc. Molecular Weight (g/mol)
1	83.1	Ethylene glycol + K^+^ − H_2_O	83.0
2	89.1	1,4-dioxane + H^+^	89.1
3	145.0	BA + OE + K^+^	145.0
4	189.1	BA + 2 OE + K^+^	189.0
5	227.1	BHEU + OE − H_2_O + H^+^	227.1
6	233.1	BA + 3 OE + K^+^ Tetraethylene glycol + K^+^	233.1 233.1
7	239.1	BA + 4 OE + H^+^	239.1
8	245.1	HEDOPEDHB + H^+^ BHEU + OE + H^+^	245.1 245.1
9	277.1	BA + 3 OE − H_2_O + H^+^ BA + 4 OE + K^+^	277.1 277.1
10	283.2	BHEU + OE + K^+^ BA + 5 OE + H^+^	283.1 283.2
11	289.2	DOPDEDHB + H^+^ HEDOPEDHB + OE + H^+^ BHEU + 2 OE + H^+^	289.1 289.1 289.1
12	321.2	BA + 5 OE + K^+^	321.1
13	327.2	DOPDEDHB + K^+^ HEDOPEDHB + OE + K^+^ BA + 6 OE + H^+^ BHEU + 2 OE + K^+^	327.1 327.1 327.2 327.2
14	365.1	BA + 6 OE + K^+^	365.1
15	371.2	DOPDEDHB + OE + K^+^ HEDOPEDHB + 2 OE + K^+^ BA + 7 OE + H^+^ BHEU + 3 OE + K^+^	371.1 371.1 371.2 371.2
16	376.3	DOPDEDHB + 2 OE BHEU + 4 OE	376.0 376.3
17	415.1	DOPDEDHB + 2 OE + K^+^ HEDOPEDHB + 3 OE + K^+^ BHEU + 4 OE + K^+^	415.1 415.1 415.1
18	421.2	DOPDEDHB + 3 OE + H^+^ HEDOPEDHB + 4 OE + H^+^ BHEU + 5 OE + H^+^	421.1 421.2 421.2
19	465.2	DOPDEDHB + 4 OE + H^+^ HEDOPEDHB + 5 OE + H^+^ BHEU + 6 OE + H^+^	465.2 465.2 465.2
20	553.2	DOPDEDHB + 6 OE + H^+^ BHEU + 8 OE + H^+^	553.3 553.3
21	773.5	DOPDEDHB + 11 OE + H^+^ BHEU + 13 OE + H^+^	773.4 773.4

where: BA, BHEU, HEDOPEDHB, and DOPDEDHB are fragmentation products of boric acid, 1,3-bis(2-hydroxyethyl)uracil, 1-(2-hydroxyethyl)-2,4-dioxopyrimidine-3-ethyl dihydroborate/3-(2-hydroxyethyl)-2,4-dioxopyrimidine-1-ethyl dihydroborate, 2,4-dioxopyrimidine-1,3-diethyl bis(dihydroborate); OE is an oxyethylene group; and the number preceding the symbol indicates the number of oxyethylene groups in a structure.

**Table 4:** The physical properties of oligoetherols.

Oligoetherol—Product of Reaction	Viscosity (N × s/m^2^) × 10^3^	Density (g/cm^3^)	Surface Tension (N/m) × 10^3^
DOPDEDHB:EC 1:12	79,500	1.25	51
DOPDEDHB:EC 1:16	192,600	1.24	52
BHEU:BA:EC 1:2:12	10,000	1.24	50
BHEU:BA:EC 1:3:12	7600	1.25	49

**Table 5:** Density, absorption of water, and linear dimension stability of selected polyurethane foams.

Composition Number (From Oligoetherol)	Density (kg/m^3^)	Absorption of Water (vol%)			Linear Dimensions Stability at Temperature 150 °C					
					Length Increase (%)		Width Increase (%)		Thickness Increase (%)	
		After 5 Mmin	After 3 h	After 24 h	After 20 h	After 40 h	After 20 h	After 40 h	After 20 h	After 40 h
2 (BHEU:BA:EC = 1:2:12)	66	4.37	9.61	27.62	3.19	4.65	3.00	3.70	2.94	4.84
4 (BHEU:BA:EC = 1:3:12)	91	4.44	11.22	18.21	0.70	0.95	0.28	0.56	−0.29	0.16
7 (DOPDEDHB:EC = 1:12)	72	3.76	7.76	18.40	2.47	3.40	1.51	2.15	4.21	5.24
9 (DOPDEDHB:EC = 1:16)	56	2.21	5.00	8.05	0.78	2.33	0.72	2.77	−1.96	1.19
AU:EC = 1:6 [19]	32	4.45	6.4	8.7	0.17	0.34	−0.61	−0.31	2.23	2.68
AU:EC:PO = 1:6:6 [20]	42	2.57	4.15	5.28	−0.29	2.03	2.36	4.72	−1.94	0

**Table 6:** Thermal resistance of selected polyurethane foams.

Composition Number (From Oligoetherol)	The Mass Loss After 30 Days Heating at Temperature (wt %)			Thermal Analysis		
	150 °C	175 °C	200 °C	T_10%_ (°C)	T_25%_ (°C)	T_50%_ (°C)
2 (BHEU:BA:EC = 1:2:12)	19	35	-	235	252	303
4 (BHEU:BA:EC = 1:3:12)	24	38	-	202	242	299
7 (DOPDEDHB:EC = 1:12)	16	34	-	228	269	322
9 (DOPDEDHB:EC = 1:16)	19	34	-	230	255	302
AU:EC = 1:6 [19]	7	23	33	260	320	550
AU:EC:PO = 1:6:6 [20]	13	29	41	235	280	330

**Table 7:** Thermal conductivity and compression strength of selected polyurethane foams.

Composition Number (From Oligoetherol)	Thermal Conductivity Coefficient Λ [W/m × K]	Compression Strength		
		Before Exposition		After Exposition at 150 °C
		σ_M_ (MPa)	ε_M_ (%)	σ_10_ (MPa)
2 (BHEU:BA:EC = 1:2:12)	0.0357	0.33	7.08	0.58
4 (BHEU:BA:EC = 1:3:12)	0.0368	0.30	7.99	0.81
7 (DOPDEDHB:EC = 1:12)	0.0357	0.29	8.33	1.12
9 (DOPDEDHB:EC = 1:16)	0.0349	0.32	9.08	1.27

σ_M_—compression strength, ε_M_—strain, σ_10_—compression strength at 10% strain.

**Table 8:** Horizontal burning tests and oxygen index of selected polyurethane foams.

Composition Number (From Oligoetherol)	Horizontal Burning Tests			Oxygen Index (vol%)
	Linear Burning Rate (mm/s)	Distance Burnt (mm)	Mass Loss During Burning (% mas.)	
2 (BHEU:BA:EC = 1:2:12)	3.42	18	4.35	22.3
4 (BHEU:BA:EC = 1:3:12)	2.06	11	3.81	24.1
7 (DOPDEDHB:EC = 1:12)	2.05	13	3.84	22.1
9 (DOPDEDHB:EC = 1:16)	2.68	20	4.97	22.0

## Data Availability

The data that support the findings of this study are available from the corresponding author upon reasonable request.
